# Supplementary material for: The causal relationship between the human gut microbiota and pyogenic arthritis: a Mendelian randomization study
Source: Front Cell Infect Microbiol. 2024 Nov 26;14:1452480. doi: 10.3389/fcimb.2024.1452480 (PMC11629706; doi:10.3389/fcimb.2024.1452480)
Supplement: Supplementary file 1 [file DataSheet1.zip › mendelian test/ebi-a-GCST90027520.csv_leave_one_out.pdf]

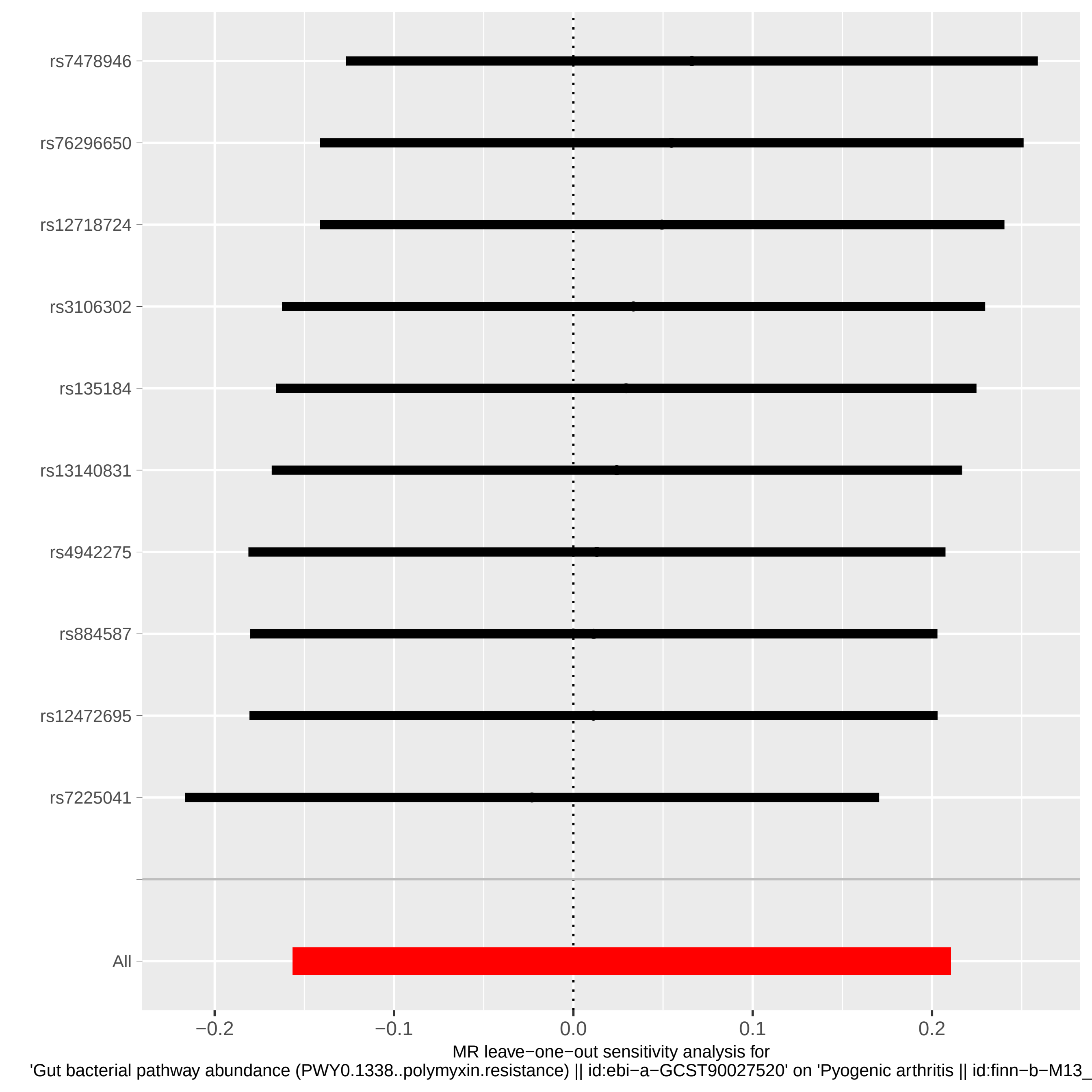
rs7478946
rs76296650
rs12718724
rs3106302
rs135184
rs13140831
rs4942275
rs884587
rs12472695
rs7225041
All
−0.2
−0.1
0.0
0.1
0.2
MR leave−one−out sensitivity analysis for
'Gut bacterial pathway abundance (PWY0.1338..polymyxin.resistance) || id:ebi−a−GCST90027520' on 'Pyogenic arthritis || id:finn−b−M13_
